# A Pilot Study Comparing Intraoral and Transcutaneous Photobiomodulation for Oral Mucositis in Head and Neck Cancer Patients Undergoing Radiotherapy or Chemoradiotherapy

**DOI:** 10.3390/jcm14072430

**Published:** 2025-04-02

**Authors:** Jordan Eber, Anna Schohn, Hélène Carinato, Youssef Brahimi, Martin Schmitt, Georges Noël

**Affiliations:** 1 Department of Radiation Oncology, Strasbourg Europe Cancer Institute (ICANS), 17 Rue Albert Calmette, BP 23025, 67033 Strasbourg, Franceg.noel@icans.eu (G.N.); 2 Department of Supportive Care in Oncology, Strasbourg Europe Cancer Institute (ICANS), 17 Rue Albert Calmette, BP 23025, 67033 Strasbourg, France; 3 Department of Medical Oncology, Strasbourg Europe Cancer Institute (ICANS), 17 Rue Albert Calmette, BP 23025, 67033 Strasbourg, France; 4 Department of Radiation Oncology, Claude Bernard Hospital-Clinic, 97 Rue Claude Bernard, 57070 Metz, France

**Keywords:** drug therapy, head and neck neoplasms, low-level laser therapy, palliative care, radiotherapy, stomatitis

## Abstract

**Background**: Photobiomodulation (PBM) therapy has shown potential in managing oral mucositis (OM), a frequent and painful side effect of radiotherapy or chemoradiotherapy in head and neck cancer patients. Although PBM is increasingly used in clinical settings, the optimal delivery method—transcutaneous or intraoral—remains undetermined. **Methods**: This prospective, single-center, randomized pilot study (clinicaltrials.gov NCT06458517) aims to compare the efficacy of transcutaneous versus intraoral PBM in preventing and managing OM in patients undergoing radiotherapy or chemoradiotherapy for cancers of the oral cavity or oropharynx. Participants will be randomized into two groups: one receiving intraoral PBM with the CareMin650™ device, and the other receiving transcutaneous PBM with the ATP38^®^ device. **Results**: Primary and secondary outcomes will include the incidence and severity of OM, treatment interruptions, patient-reported pain levels, and quality of life, assessed using validated tools. **Conclusions**: This study will provide comparative data on two PBM modalities, contributing to the development of standardized PBM protocols in supportive oncology care and informing future multicenter trials aimed at improving patient outcomes during radiotherapy for head and neck cancer.

## 1. Introduction

Head and neck cancers account for approximately 4–5% of all malignancies worldwide, with over 900,000 new cases annually. These cancers comprise a heterogeneous group of tumors arising in the upper aerodigestive tract, including the oral cavity, oropharynx, hypopharynx, nasopharynx, and larynx. The predominant histological type is squamous cell carcinoma, representing more than 90% of head and neck cancers [1]. Additional subtypes include salivary gland tumors, which are histologically diverse and relatively rare, and non-melanoma skin cancers of the head and neck region, which are common but often underreported.

In 2020, lip and oral cavity cancers accounted for approximately 389,846 new cases and 186,427 deaths globally, with particularly high incidence rates in South and Southeast Asia [2]. Oropharyngeal cancers represented over 106,000 cases and 52,000 deaths, with tobacco, alcohol, and HPV infection being key risk factors [3]. Nasopharyngeal cancers, although geographically concentrated in East and Southeast Asia, contributed to more than 120,000 new cases and 73,000 deaths worldwide [4]. Hypopharyngeal cancers, though less common, remain among the most lethal, with over 86,000 new cases and a high mortality-to-incidence ratio [2]. Salivary gland cancers accounted for approximately 55,000 new cases globally, with variable histology and outcomes depending on subtype and stage at diagnosis [4].

These cancers are frequently diagnosed at advanced stages, often due to late-onset symptoms and limited accessibility of certain anatomical sites. Consequently, patients commonly require multimodal treatment strategies, including surgery combined with radiotherapy and/or chemotherapy, either as neoadjuvant, adjuvant, or concomitant therapy. However, these therapies are often associated with debilitating side effects, among which oral mucositis (OM) is one of the most frequent and severe complications [5,6].

### 1.1. Oral Mucositis: A Major Clinical Challenge

OM is an acute inflammatory condition that affects 59–100% of patients receiving radiotherapy for oral cavity and oropharyngeal cancers, leading to painful ulcerative lesions that impair swallowing, nutrition, and overall quality of life [7,8]. Severe cases can result in treatment delays or dose reductions, which may compromise oncological outcomes [9,10,11,12]. Current treatment options, such as topical agents (e.g., medicated mouthwashes) and systemic analgesics, provide suboptimal relief, highlighting the urgent need for more effective strategies [13,14].

Several alternative or adjunctive strategies have been investigated for the management of OM in cancer patients. Histatin-5 mucoadhesive gel, a salivary antimicrobial peptide formulation, has shown promising results in preclinical and in vivo studies for promoting mucosal healing [15]. Bioadhesive agents, which form protective films over ulcerated mucosa, have also demonstrated efficacy in reducing mucositis severity and improving patient comfort, as confirmed by a recent systematic review and meta-analysis [16]. Furthermore, herbal medicine is being explored as a supportive care option to alleviate radiation-induced mucosal toxicity, particularly in traditional Asian medicine contexts [17]. At the molecular level, the keratinocyte growth factor (KGF)/KGF receptor (KGFR) pathway has been identified as a dual therapeutic target for both epithelial repair and cancer regulation, offering potential in mucosal protection strategies during cancer treatment [18].

These approaches complement light-based therapies, which remain among the most extensively studied interventions for mucositis prevention and treatment in oncology [10].

Radiation-induced OM is a multi-phase pathological process triggered by direct cytotoxic damage to epithelial cells, leading to inflammatory cascades, reactive oxygen species generation, and further tissue breakdown. Predictive modeling has identified key risk factors for severe OM, including treatment acceleration, mean radiation dose to the oral cavity, smoking status, and patient gender [19,20].

Given the complexity of OM pathogenesis and its profound impact on patient outcomes, novel therapeutic approaches are warranted.

### 1.2. Photobiomodulation Therapy: A Promising Approach

Photobiomodulation (PBM), the application of low-level light therapy, has emerged as an innovative and promising approach for OM prevention and management. Recent evidence supports its ability to reduce inflammation, accelerate tissue healing, and alleviate pain without significant adverse effects [10,21,22,23].

However, clinical implementation remains inconsistent, primarily due to a lack of standardized protocols. PBM can be delivered using two main approaches:

- Intraoral PBM, where light is applied directly to the affected mucosa, ensuring targeted irradiation.
- Transcutaneous PBM, a non-invasive method where light is applied externally through the skin, though tissue penetration may be a limiting factor [24].

Several clinical trials have demonstrated the efficacy of PBM in reducing OM severity and improving patient outcomes. A phase II trial reported that prophylactic PBM significantly lowered the incidence of severe OM in patients undergoing concurrent chemoradiation, with a severe OM rate of only 23%, markedly lower than historical controls [25]. Additionally, preconditioning with PBM before chemotherapy has been shown to prevent mucositis onset in up to 86.7% of patients, further highlighting its role in improving quality of life and treatment adherence [26].

Moreover, emerging evidence suggests that PBM may not only alleviate OM symptoms but also contribute to improved survival rates. Some studies indicate a significant correlation between the number of PBM sessions and increased overall survival in patients undergoing chemoradiation [27].

Despite these promising findings, concerns regarding PBM’s potential effects on tumor progression persist. However, recent research has suggested that PBM does not compromise oncological outcomes and may even enhance the immune response during cancer treatment [23]. This has led the World Association for Photobiomodulation Therapy (WALT) to establish evidence-based recommendations supporting PBM for OM management and emphasizing the need for standardized treatment protocols [28].

While systematic reviews and meta-analyses generally support the efficacy of PBM, a recent umbrella review found that the overall quality of evidence remained low, underscoring the necessity for high-quality randomized controlled trials to refine PBM protocols and confirm its benefits [13]. Despite these promising findings, PBM implementation in clinical practice remains inconsistent, with no consensus on the optimal treatment parameters, including the choice between transcutaneous and intraoral delivery methods [29].

### 1.3. Justification for the Comparison Between Intraoral and Transcutaneous PBM

PBM relies on the application of low-intensity light to stimulate biological processes, particularly the reduction in inflammation and the acceleration of tissue repair. The effectiveness of this approach is influenced by several parameters, including wavelength, applied dose, and the depth of light penetration in biological tissues. Studies on light penetration in both intraoral and extraoral tissues have demonstrated that wavelengths in the red spectrum (600–700 nm) and near-infrared spectrum (700–1000 nm) exhibit different penetration capacities depending on the medium they traverse. Intraoral PBM, applied directly to the oral mucosa, allows for the efficient transmission of photon energy to the epithelial and subepithelial layers, thereby maximizing local bio-stimulation [21]. Additionally, studies have shown that the extraoral application of near-infrared phototherapy can significantly reduce mucositis-related pain in patients undergoing hematopoietic stem cell transplantation, suggesting that transcutaneous PBM may be effective despite potential energy loss due to tissue absorption [30].

A study by Jagdeo et al. evaluated light diffusion through various thicknesses of human tissues and demonstrated that photon intensity decreased exponentially with depth, particularly in highly vascularized and dense tissues [31]. This suggests that intraoral PBM may have a significant advantage in directly targeting mucosal lesions, whereas transcutaneous PBM may be more suitable for deeper or less accessible areas (Huang et al., 2009) [32]. Bensadoun et al. highlighted that while both intraoral and extraoral PBM approaches were effective for managing OM, intraoral PBM ensured the direct irradiation of mucosal tissues, optimizing energy absorption and therapeutic efficacy [22]. In contrast, extraoral PBM, particularly with near-infrared wavelengths, may reach intraoral structures indirectly but is subject to potential energy loss due to absorption and scattering by intermediate tissues.

Recent clinical and preclinical studies have further explored the efficacy of both approaches, highlighting the need for a direct comparative analysis. Ramos-Pinto et al. conducted a randomized clinical trial in hematopoietic stem cell transplant patients and found that intraoral and extraoral PBM were both effective in reducing mucositis severity, with intraoral PBM providing a more targeted effect [33]. Similarly, Thieme et al. demonstrated in a chemotherapy-induced mucositis animal model that intraoral PBM promoted faster healing, whereas extraoral PBM, despite being less direct, still contributed to mucosal recovery [34]. Adnan et al. emphasized the need for optimized dosimetric parameters in extraoral PBM to ensure sufficient photon penetration and therapeutic effectiveness [35]. These findings suggest that while intraoral PBM may offer superior localized effects, extraoral PBM remains a promising alternative, particularly when intraoral application is not feasible.

This randomized pilot study aims to compare the efficacy of transcutaneous and intraoral PBM in preventing and managing OM in patients undergoing radiotherapy for oral cavity and oropharyngeal cancers. By addressing gaps in clinical practice, this study seeks to provide evidence that can contribute to the standardization of PBM protocols and ultimately enhance patient care outcomes

## 2. Materials and Methods

### 2.1. Study Setting

This randomized pilot study will be conducted at the Institut de Cancérologie Strasbourg Europe (ICANS), France, in the University Department of Radiotherapy and the Department of Oncology Supportive Care. The study will aim to compare the efficacy of transcutaneous and intraoral PBM in preventing and managing OM in patients receiving radiotherapy for cancers of the oral cavity and oropharynx. The study will follow a randomized 1:1 allocation of patients to either the transcutaneous PBM or intraoral PBM group, with stratification based on tumor location, prior surgery, and chemotherapy regimen.

This study was registered on clinicaltrials.gov in November 2024 (NCT06458517).

### 2.2. Inclusion and Exclusion Criteria

For a full overview of all inclusion and exclusion criteria, see Table 1.

### 2.3. PBM Protocols

#### 2.3.1. Transcutaneous PBM

Patients will receive transcutaneous PBM using the ATP38^®^ device (Swiss Bio Inov S.A., Moudon, Switzerland). They will be either seated or lying down during the procedure. Three light-emitting panels will be positioned around the patient’s oral cavity—one in front of the lips and two on each side of the cheeks—by a State Registered Nurse or a Medical Electroradiology Technician. Protective glasses will be provided for the duration of the session. The device will emit infrared light ranging from 600 to 820 nm, a wavelength range selected for its ability to effectively penetrate soft tissues while minimizing absorption by superficial structures, thereby allowing deeper energy delivery to the oral mucosa [35]. The power density of 2 J/cm^2^ for preventive treatment and 6 J/cm^2^ for curative treatment aligns with previous studies demonstrating optimal tissue stimulation and anti-inflammatory effects within this fluence range while avoiding potential photoinhibition effects at higher doses [21].

The average irradiation duration of 240 s for preventive therapy and 720 s for curative therapy will ensure that the total energy delivered per session remains within the recommended range for effective PBM while maintaining a practical treatment duration for clinical application [33]. Sessions will be administered twice weekly as a preventive measure and three times weekly if OM ≥ grade 1 develops, following prior clinical trials that demonstrated a significant reduction in mucositis severity with a similar treatment frequency [36].

#### 2.3.2. Intraoral PBM

Patients will receive intraoral PBM using the CareMin650™ device (NeoMedLight, Villeurbanne, France), with two intraoral pads (CareMin650™ Oral Pad) positioned within the oral cavity while seated. The device will emit red light at 650 nm, a wavelength chosen for its superior absorption by epithelial and subepithelial tissues, maximizing local bio-stimulation [21]. The power density of 3 J/cm^2^ for preventive treatment and 6 J/cm^2^ for curative treatment is based on evidence indicating that this fluence range effectively promotes wound healing and reduces inflammation while remaining within the safety threshold for oral tissues [35]. The average irradiation duration of 138 s for preventive therapy and 260 s for curative therapy was selected to ensure an appropriate energy dose while minimizing patient discomfort [33]. The procedure will be performed by a State Registered Nurse or a Medical Electroradiology Technician, ensuring strict adherence to the pain-free application rule when placing the oral pads. The treatment schedule will follow the same protocol as that of transcutaneous PBM.

### 2.4. Standard Supportive Care Measures

All patients enrolled in this study will receive standard preventive and therapeutic care for OM, in accordance with established national and international oncology guidelines. Preventive strategies will include systematic oral hygiene protocols, routine dental evaluations, the use of bicarbonate-based mouth rinses, and recommendations for adequate hydration. These measures will be implemented uniformly across both treatment groups. In the event of OM development, patients will be managed according to clinical needs using standard interventions such as topical analgesics, mucosal protectants, and systemic analgesia, following institutional supportive care protocols.

The use of any concomitant treatments—including pharmacological agents (e.g., antifungals, corticosteroid-based rinses, local anesthetics), dietary modifications, or additional supportive measures—will be systematically recorded for each patient throughout the study. This approach will ensure both ethical patient care and the ability to account for potential confounding factors when evaluating the effects of intraoral versus transcutaneous PBM.

### 2.5. Data Collection

Data will be collected from electronic medical records and will include demographics, clinical history, treatment details, and outcomes. Weekly evaluations of OM will be performed by clinicians independent from PBM administration and unaware of group allocation using National Cancer Institute Common Terminology Criteria for Adverse Events (NCI-CTCAE) v5.0 criteria. Additional data will include visual analog scale pain scores, the quality of life questionnaires European Organisation for Research and Treatment of Cancer Quality of Life Questionnaire–Core 30 (EORTC QLQ-C30) and European Organisation for Research and Treatment of Cancer Quality of Life Questionnaire–Head and Neck Module (EORTC QLQ-HN35) [37,38], and nutritional intake using the Simple Evaluation of Food Intake (SEFI) tool.

### 2.6. Statistical Analysis

Descriptive statistics will summarize the patient demographics and clinical characteristics. Quantitative variables will be expressed as mean ± standard deviation, while categorical variables will be presented as frequencies and percentages. Between-group comparisons will be conducted using appropriate statistical tests, including t-tests for continuous data and chi-squared tests for categorical data. A significance level of *p* < 0.05 will be considered statistically significant.

### 2.7. Ethical Approval

This study will be conducted in accordance with the Declaration of Helsinki and was reviewed and approved by the Protection of persons Committee (CPP) Île-de-France, under the national registration number 2023-A02113-42 (internal reference: 2022-018, SI file number: 24.01626.000309). Authorization was also granted by the French National Agency for the Safety of Medicines and Health Products (ANSM) on 19 April 2024, under the same national IDRCB number (2023-A02113-42), as a Category 4.2 clinical investigation. The sponsor of the study is the Institut de Cancérologie Strasbourg Europe (ICANS). All participants will provide written informed consent prior to inclusion in the study.

### 2.8. Objectives

#### 2.8.1. Primary Endpoint

The primary outcome measure of this study is the reduction in the incidence of OM (grade ≥ 2) in patients undergoing radiotherapy or chemoradiotherapy for cancers of the oral cavity or oropharynx. The comparison will be based on the method of PBM delivery: transcutaneous versus intraoral.

#### 2.8.2. Secondary Endpoints

- Treatment interruptions: comparison of the number and duration of treatment interruptions (radiotherapy or chemoradiotherapy), exceeding or below 7 days, between the two PBM delivery methods.
- Mucositis duration: comparison of the total duration of OM (all grades) between transcutaneous and intraoral PBM methods.
- Unplanned hospitalizations: evaluation of the number of unplanned hospitalizations during treatment in both groups.
- Nutritional support: comparison of the time to initiation and frequency of artificial nutrition (enteral or parenteral) between the two PBM groups.
- Pain and analgesic use: assessment of differences in patient-reported pain levels and analgesic consumption between the two PBM methods.
- Quality of life: evaluation of the impact of PBM methods on patients’ quality of life.
- Infection rates: comparison of local and systemic infection rates, graded by NCI-CTCAE v5.0, between the two groups.
- Xerostomia: evaluation of xerostomia incidence and severity (all grades) in patients treated with each PBM method.

### 2.9. Participant Timeline

**Figure 1:**
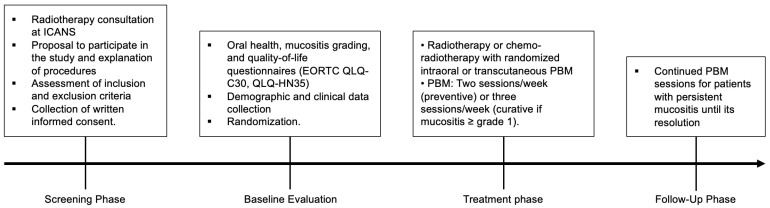
RADIO-PBM trial schema. Abbreviations: PBM: photobiomodulation; EORTC QLQ-C30, European Organisation for Research and Treatment of Cancer Quality of Life Questionnaire–Core 30; QLQ-HN35, European Organisation for Research and Treatment of Cancer Quality of Life Questionnaire–Head and Neck Module.

Figure 1 shows the different stages of the clinical trial.

### 2.10. Project Duration and Expected Outcomes

#### 2.10.1. Project Duration

The study is expected to span a total duration of 20 months, including an 18-month recruitment period and a 2-month follow-up phase for each participant. The investigation will begin on 1 February 2025, and conclude on 1 October 2026. This timeline will allow sufficient time for patient enrollment, data collection, and initial analysis.

#### 2.10.2. Expected Outcomes

The study aims to generate robust comparative data on the efficacy of transcutaneous versus intraoral PBM in the prevention and management of OM among patients undergoing radiotherapy or chemoradiotherapy. By addressing this critical aspect of supportive care, the findings are expected to contribute significantly to the optimization of treatment outcomes and patient well-being.

One of the primary outcomes anticipated is the identification of the most effective PBM delivery method in reducing the incidence, severity, and duration of OM. This insight will not only enhance clinical understanding but also support the adoption of tailored approaches to mitigate the burden of this common complication. Additionally, the study is expected to shed light on PBM’s broader impact on patient care, particularly in terms of reducing treatment interruptions, minimizing unplanned hospitalizations, and improving pain management and quality of life.

Furthermore, the results are likely to inform the development of standardized, evidence-based protocols for PBM application in clinical settings, ensuring consistent and effective use of this therapy across diverse patient populations. Lastly, the study will provide foundational data for future research, serving as a basis for larger multicenter trials and long-term evaluations of PBM’s efficacy and safety.

Ultimately, these findings are expected to bridge current gaps in clinical practice, paving the way for improved supportive care strategies for patients undergoing radiotherapy for head and neck cancers. By achieving these outcomes, the study will contribute to both advancing scientific knowledge and enhancing patient outcomes in this challenging treatment context.

## 3. Discussion

OM is a frequent and debilitating side effect of radiotherapy and chemoradiotherapy in patients with head and neck cancer, leading to significant impairments in nutritional intake, treatment continuity, and overall quality of life. Despite advancements in supportive care, existing preventive measures remain limited in efficacy [14].

PBM therapy has gained increasing recognition as an effective non-pharmacological intervention for OM. Its mechanisms—including reduction in inflammation, promotion of epithelial healing, and analgesic effects—have been well documented [21,22]. In 2019, MASCC/ISOO published guidelines recognizing PBM as a recommended intervention for the prevention of OM in various cancer populations [10,21].

Several randomized controlled trials have confirmed PBM’s efficacy [39,40,41,42,43,44]. Gouvêa De Lima et al. demonstrated that intraoral low-level laser therapy significantly reduced the incidence and severity of OM in head and neck cancer patients receiving concurrent chemoradiotherapy [45]. A recent meta-analysis with a trial sequential analysis confirmed the efficacy of PBM in reducing the severity and duration of chemotherapy-induced OM, providing conclusive evidence with moderate certainty. Khalil et al. further reported that PBM preconditioning not only decreased OM incidence but also improved quality of life in adult chemotherapy patients [26]. These results collectively support PBM’s clinical relevance in oncology supportive care.

However, while intraoral PBM has shown robust efficacy, its tolerability and logistical constraints (e.g., need for direct contact, patient discomfort) have prompted interest in alternative approaches such as transcutaneous PBM. A randomized controlled trial compared intraoral versus extraoral PBM in hematopoietic stem cell transplant recipients, reporting similar effectiveness in OM prevention, though intraoral PBM provided more localized therapeutic effects [33].

Additionally, Zecha et al. highlighted the need for well-defined safety parameters in PBM application through their systematic review on laser therapy, underscoring the importance of optimizing wavelength, dose, and delivery technique [10,21].

The present study addresses this clinical gap by directly comparing intraoral and transcutaneous PBM delivery methods using standardized protocols in head and neck cancer patients receiving (chemo)radiotherapy. The study’s strengths include its prospective randomized design, the use of clearly defined PBM parameters for both modalities, and a comprehensive evaluation of outcomes including OM incidence, pain levels, treatment interruptions, nutritional support, and quality of life. However, limitations include its single-center design and relatively small sample size, typical of pilot studies. Additionally, the lack of a third arm without PBM limits the interpretation of absolute efficacy. Nevertheless, the comparative design offers critical insights into the relative benefits and feasibility of each PBM modality in a real-world clinical setting.

Future studies should expand upon these findings by incorporating larger, multicenter cohorts and extended follow-up periods. Such follow-up is essential not only to confirm sustained benefits but also to monitor for potential long-term adverse effects of PBM, such as the risk of secondary malignancies in the oral cavity. Bezinelli et al. conducted a 15-year retrospective study investigating different PBM protocols for OM control in hematopoietic cell transplantation patients [46]. They found no immediate adverse effects or secondary malignancies associated with PBM, suggesting the long-term safety of the therapy. However, further research with extended follow-up is necessary to corroborate these findings and ensure the comprehensive safety profile of PBM in oncology supportive care.

## 4. Conclusions

This study will evaluate the effectiveness of intraoral and transcutaneous PBM in managing OM during radiotherapy or chemoradiotherapy for head and neck cancers. By directly comparing these two PBM delivery methods, our findings aim to establish evidence-based protocols that optimize OM prevention and treatment. Specifically, this study will investigate whether intraoral PBM, which delivers targeted irradiation to affected tissues, provides superior therapeutic outcomes compared to transcutaneous PBM, which offers a non-invasive alternative.

By contributing to the standardization of PBM protocols in oncology, this study seeks to enhance clinical consistency and improve patient care. The primary objectives are to reduce mucositis severity, minimize treatment interruptions, and enhance patient-reported quality of life, ultimately facilitating the broader integration of PBM into routine oncology practice.

Despite its strengths, this study has limitations, including its single-center design and limited sample size, which may impact the generalizability of the results. Multicenter trials with larger patient cohorts will be essential to validate these findings and refine PBM treatment parameters for widespread clinical adoption.

Future research should focus on optimizing PBM dosimetry, further exploring its molecular and immunological mechanisms of action, and integrating personalized treatment approaches based on patient-specific factors. Addressing these aspects will advance PBM as a standardized, effective supportive care strategy in oncology, ultimately improving treatment tolerability, reducing complications, and enhancing quality of life for patients undergoing radiotherapy for head and neck cancers.

## Figures and Tables

**Table 1:** Inclusion and exclusion criteria.

Inclusion Criteria	Exclusion Criteria
Adult patients diagnosed with cancer of the oral cavity or oropharynx. Scheduled to receive radiotherapy or chemoradiotherapy. Karnofsky Performance Status (KPS) > 60%. Fluent in French and able to provide informed consent. Enrolled in the French national healthcare system.	Allergy to polyurethane or contraindications to PBM. Prior irradiation to the head and neck region. Pregnant or lactating women. Presence of medical devices such as pacemakers or epilepsy.

## Data Availability

Not applicable.

## Abbreviations

The following abbreviations are used in this manuscript:

PBM	Photobiomodulation
OM	Oral mucositis
KPS	Karnofsky Performance Status
SEFI	Simple Evaluation of Food Intake
